# Fractures, Bone Mineral Density, and Final Height in Craniopharyngioma Patients with a Follow-up of 16 Years

**DOI:** 10.1210/clinem/dgz279

**Published:** 2020-03-07

**Authors:** Selveta S van Santen, Daniel S Olsson, Marry M van den Heuvel-Eibrink, Mark Wijnen, Casper Hammarstrand, Joseph A M J L Janssen, Gudmundur Johannsson, Aart J van der Lely, Sebastian J C M M Neggers

**Affiliations:** 1 Department of Medicine, Endocrinology; Erasmus Medical Center, GD Rotterdam, The Netherlands; 2 Department of Paediatric Oncology/Haematology, Erasmus MC – Sophia Children’s Hospital, CN Rotterdam, The Netherlands; 3 Princess Máxima Center for Paediatric Oncology, CS Utrecht, The Netherlands; 4 Department of Medicine, Endocrinology; Sahlgrenska University Hospital, Gothenburg, Sweden; 5 Department of Internal Medicine and Clinical Nutrition, Institute of Medicine, Sahlgrenska Academy, University of Gothenburg, Gothenburg, Sweden

**Keywords:** craniopharyngioma, bone health, fractures, bone mineral density, final height

## Abstract

**Context:**

Pituitary hormonal deficiencies in patients with craniopharyngioma may impair their bone health.

**Objective:**

To investigate bone health in patients with craniopharyngioma.

**Design:**

Retrospective cross-sectional study.

**Setting:**

Dutch and Swedish referral centers.

**Patients:**

Patients with craniopharyngioma (n = 177) with available data on bone health after a median follow-up of 16 years (range, 1-62) were included (106 [60%] Dutch, 93 [53%] male, 84 [48%] childhood-onset disease).

**Main outcome measures:**

Fractures, dual X-ray absorptiometry-derived bone mineral density (BMD), and final height were evaluated. Low BMD was defined as T- or Z-score ≤-1 and very low BMD as ≤-2.5 or ≤-2.0, respectively.

**Results:**

Fractures occurred in 31 patients (18%) and were more frequent in men than in women (26% vs. 8%, *P* = .002). Mean BMD was normal (Z-score total body 0.1 [range, -4.1 to 3.5]) but T- or Z-score ≤-1 occurred in 47 (50%) patients and T-score ≤-2.5 or Z-score ≤-2.0 in 22 (24%) patients. Men received less often treatment for low BMD than women (7% vs. 18%, *P* = .02). Female sex (OR 0.3, *P* = .004) and surgery (odds ratio [OR], 0.2; *P* = .01) were both independent protective factors for fractures, whereas antiepileptic medication was a risk factor (OR, 3.6; *P* = .03), whereas T-score ≤-2.5 or Z-score ≤-2.0 was not (OR, 2.1; *P* = .21). Mean final height was normal and did not differ between men and women, or adulthood and childhood-onset patients.

**Conclusions:**

Men with craniopharyngioma are at higher risk than women for fractures. In patients with craniopharyngioma, a very low BMD (T-score ≤-2.5 or Z-score ≤-2.0) seems not to be a good predictor for fracture risk.

Craniopharyngioma is a rare tumor in the pituitary region with peak incidences in the age categories 5 to 9 and 40 to 44 years (1). Age of onset correlates highly with tumor histology: the adamantinomatous subtype occurs mainly in childhood, and in adulthood, while the papillary subtype almost exclusively occurs at adult age (2). The pathogenesis of the 2 subtypes differs as well because adamantinomatous craniopharyngioma is driven by somatic mutations in CTNNB1, whereas the papillary subtypes harbor BRAF^V600E^ mutations (2). Craniopharyngioma patients (CP) require extensive long-term follow-up because the survival rate is relatively high and the patients often suffer from multiple comorbidities (1, 3). Several comorbidities could give CP an increased risk of fractures, osteoporosis, and reduced growth: CP are at risk of pituitary insufficiency and/or possibly nonphysiological replacement therapy (3–9), visual deficiency (10), late puberty induction, hypothalamic damage, and limited physical activity (11–14). In addition, chronic neurological disease is associated with clumsiness and high incidence of falls (15, 16). Moreover, antiepileptic therapy may even contribute more to the lifetime fracture risk than the seizures themselves (17). All of these factors may influence bone health and could result in osteoporosis. Osteoporosis is a systemic disease characterized by 3 elements: loss of bone mass, deterioration of the microarchitecture of the bone, and an increased fracture risk (18). Fractures may cause chronic pain, disability, and need for rehabilitation (18).

Despite the knowledge of these frequent occurring comorbidities, studies addressing fracture risk and osteoporosis or bone mineral density (BMD) in CP are scarce. Previously found frequencies of fractures were higher compared with matched populations and lie between 15% and 25% (19–21). Olsson et al. described an increased risk of fractures with a standardized incidence rate of 2.1 in a large cohort of Swedish CP (1). No studies have yet extensively investigated risk factors for fractures in CP.

In foregoing research regarding BMD, very few adulthood-onset (AO) patients were included: osteoporotic/osteopenic BMD values were found in 6 of 10 and 5 of 6 cases, respectively (22, 23). The studies regarding childhood-onset (CO) CP are slightly larger but led to different conclusions regarding the influence of gender on the risk of fracture (11, 24); Müller et al. found that men were at risk for lower volumetric BMD (n = 61) (24), whereas Holmer et al. found a low BMD in women (n = 39) (11).

Only a few studies have addressed final height, mainly in CO CP. In these studies, CO CP reach slightly below average or normal final height (19, 25–33).

In summary, information on bone health in CP is scarce and contradictory. Also, factors predicting increased risk of fractures in patients with CP have not yet been studied. To improve long-term care and increase knowledge of possible risk factors of impaired bone health in CP, we investigated bone fractures, BMD, and final height in a large CP cohort.

## Design and Methods

In this retrospective cross-sectional study, CO and AO CP treated at the Erasmus University Medical Center (Rotterdam, the Netherlands)/Sophia Children’s Hospital (Rotterdam, the Netherlands), and Sahlgrenska University Hospital (Gothenburg, Sweden) were included. Data were collected as previously described (10, 34). Subjects were included if data were available on fractures, dual X-ray absorptiometry (DXA) scan results for BMD, or on final height.

Data on fractures were acquired as described previously (10); data on fractures were included from 1987 until April 2019. Data regarding fractures from the Dutch patients concern both inpatient and outpatient data; for the Swedish patients, data until 2014 concern only inpatient data. If only the year of fracture was known, the first of January was used as date to calculate age at fracture. Medication to improve BMD was defined as current or past use of bisphosphonates, vitamin D, or calcium. Gonadal axis replacement therapy or other hormonal replacement therapy was not included because the indication of treating pituitary deficiencies was broader than only improving BMD and not solely for preservation of bone health. The definition of hypothalamic damage was described before: it concerns tumor- and/or treatment-related injury to the hypothalamus and/or third ventricle as visualized by neuroimaging (34). The research proposal was accepted by the Ethical Review Board of the Erasmus Medical Center and the Regional Ethical Review Board of the Gothenborg University; all patients gave informed consent.

All types of DXA scanners were accepted in the analysis and include Lunar DPXL, Lunar iDXA, and Lunar Prodigy. A list of accepted DXA scanners is shown in all Supplementary Tables are available at DANS online digital research repository (35). For the current study, low BMD was defined as T- or Z-score below -1. Osteoporosis was defined as T-score below -2.5 or Z-score below -2. Osteopenia was defined as T- or Z-score between -1 and -2.5 or -2, respectively. If it is not specified in the text whether low BMD, osteopenia, or osteoporosis are based on T- or Z-score, or on which site, it was found to exceed the limit of either T- or Z-score, and was measured at the location of femur neck, L2-L4, and/or total body (36). If multiple DXA scans were performed, the most recent was chosen. The decision to perform a DXA scan was made clinically by the physician treating the patient.

Final height was defined as the highest measured value, after the age of 18 years. Standardized deviation scores (SDS) or final height for Dutch subjects were calculated based on requested data with references from the Dutch National Statistics (“Centraal Bureau voor Statistiek”) (37) based on age, sex, and year of measurement (available on request).

If final height was measured before 1981 or after 2017, references of 1981 or 2017, respectively, were used in Dutch subjects. Final height SDS of Swedish subjects was calculated with references of the same sex compared with young adults with birth year matched as closely as possible (38–40).

### Statistical analysis

Statistical analysis was performed with IBM SPSS Statistics for Windows, version 24.0, and GraphPad, version 8.01. Data are presented as mean and SD unless stated otherwise. Significance was accepted if *P ≤ *.05. For normally distributed data, an unpaired *t*-test was used for group comparisons, whereas a Mann-Whitney *U* test was used for nonnormally distributed data. Comparisons of proportions were conducted using either the χ ^2^ test or the Fisher exact test as indicated; in case of comparison of 2 related dichotomous variables, McNemar test was applied. To identify influencing factors of low BMD at last DXA scan and fractures, a univariable logistic regression model was primarily performed; only significant predictors (*P* < .05) were entered in the multiple logistic regression analysis thereafter. If the number of events allowed it, the multiple logistic regression model was refined by adding variables with *P* < .20. If more variables were significant in univariable analysis than could be entered in multivariable model because of the size of the cohort, the most significant variables were selected in multivariable analysis. If the options were comparable, the variable contributing to the best discrimination and highest Nagelkerke *R*^2^ was chosen. The dependent variables were studied with forced entry. For fractures, a Cox regression model was established with the apparent main predictors from the logistic regression model. The assumption of proportional hazards was verified by a goodness-of-fit test with Schoenfeld residuals and time and checking significance of time-dependent covariates. The Wilcoxon rank sum test was used to compare nonnormally distributed variables and the Student *t*-test was used for normally distributed variables. A 2-way ANOVA was applied for comparisons of multiple groups. The incidence rate was calculated as total incidence of fractures divided by the cumulative follow-up years and multiplied by 1000 to calculate the fracture rate per 1000 person-years.

## Results

### Baseline characteristics

Baseline characteristics of the 177 CP are depicted in Table 1 and (35). Data were available on DXA scan results for 117 patients and on final height for 171 patients. Patients with a DXA scan available had a higher percentage of diabetes insipidus (68% vs. 51%, *P* = .03), of growth hormone deficiency (GHD) (93% vs. 72%, *P* < .001) and of TSH deficiency (95% vs. 86%, *P* = .06) than patients with no DXA scan, and less often epilepsy (13% vs. 27%, *P* = .03) or a hydrocephalus (23% vs. 40%, *P* = .03). There was no difference in fractures (18% vs. 18%, *P* = .95). The cohort consisted of 93 AO CP (53%). There were 106 Dutch (60%) and 71 Swedish (40%) patients included; 93 patients were men (53%) and 15 patients (8%) used medication for epilepsy. The median age at last follow-up was 45 years (range, 15-92). Median follow-up time was 16 years (range, 1-62). Mean body mass index (BMI) was 31 ± 7 kg/m^2^ (range, 17-60) in all patients at last DXA scan (or, if DXA scans at adult age were unavailable, last available BMI) and was higher in women than in men (30 ± 6 vs. 33 ± 8 kg/m^2^, *P* = .02). CO CP had more often received radiotherapy as tumor treatment (66% vs. 38%, *P* < .001), had a higher occurrence of hydrocephalus (38% vs. 17%, *P* = .002) and epilepsy (26% vs. 12%, *P* = .01) than AO CP. In addition, CO CP had more often a GHD (92% vs. 79%, *P* = .02) and had higher occurrence of diabetes insipidus (77% vs. 52%, *P* < .001). Sex hormone replacement therapy was used in 125 patients (94% of women aged 50 years or younger and men with gonadal axis failure). In 2 patients, presence of GHD was unknown. In the GHD group, CO CP were more often using GH replacement therapy (GHRT) than AO CP (87% vs. 74%, *P* = .04). Five patients (3%) underwent bariatric surgery (1 gastric sleeve, 4 gastric bypass); all of them were female. Of the 10 patients who were not treated with surgery, 5 had yttrium, 1 was initially treated with radiation therapy, 1 had cyst aspiration, and the others were closely followed with imaging without therapy. The nonsurgery group had significant lower frequency of diabetes insipidus (10% vs. 67% in the entire cohort, *P* < .001), TSH deficiency (50% vs. 95%, *P* < .001), and trend toward lower visual deficiencies (50% vs. 78%, *P* = .09). Furthermore, BMI was not significantly different (*P* = .34). The tumor was described as stable during follow-up of these patients.

**Table 1.** Baseline Characteristics of the Included CP Survivors

		All Survivors (n = 177)	Males (n = 93)	Females (n = 84)	*P* Value
Age at last follow-up, y^a^		45 (15–92)	45 (16–92)	45 (15–82)	.83
Age at presentation, y^a^		23 (0–79)	26 (0–79)	22 (4–73)	.92
(Female/corresponding) gender, n (%)		84 (48%)	93 (53%)	84 (47%)	.50
Childhood onset disease, n (%)		84 (48%)	44 (46%)	40 (48%)	.97
Tumor location at presentation, n (%)	Intrasellar	6 (4%)	5 (5%)	1 (1%)	.22
	Suprasellar	69 (41%)	35 (52%)	34 (44%)	.57
	Intra-/suprasellar	95 (56%)	52 (57%)	43 (55%)	.86
Craniopharyngioma treatment	Surgery only	77 (44%)	42 (46%)	35 (42%)	.55
	Radiation only	2 (1%)	1 (1%)	1 (1%)	1.00
	Surgery and radiation	88 (50%)	44 (48%)	44 (52%)	.59
	^90^Yttrium brachytherapy	23 (13%)	8 (9%)	15 (18%)	.09
Pituitary deficiencies	GH deficiency^b^	149 (85%)/120 (81%)	78 (86%)	71 (85%)	.83
	TSH deficiency^a^	162 (92%)/161 (99%)	88 (95%)	74 (88%)	.12
	Hypogonadism^b^	155 (88%)/125 (94%)	84 (90%)	71 (85%)	.24
	Corticotrophic deficiency^b^	146 (83%)/142 (99%)	81 (87%)	65 (77%)	.09
	ADH deficiency^b^	113 (64%)/111 (98%)	61 (66%)	52 (61%)	.61
Medical history	Recurrence/progression	67 (39%)	33 (36%)	34 (41%)	.53
	Hypothalamic damage	63 (40%)	35 (42%)	28 (38%)	.67
	Hydrocephalus ever	47 (27%)	22 (24%)	25 (30%)	.38
	Visual impairment	125 (77%)	67 (41%)	58 (36%)	.50
	Epilepsy	33 (19%)	18 (19%)	15 (18%)	.80
	Diabetes mellitus	26 (15%)	11 (12%)	15 (18%)	.26
	BMD medication	21 (12%)	6 (7%)	15 (18%)	**.02**

In 7 patients, tumor location at presentation was unknown. Bold values represent significant differences between males and females (*P* ≤ .05).

^a^Median (range).

^b^All/using replacement therapy.

Abbreviations: ADH, antidiuretic hormone; BMD medication, bone mineral density-increasing medication.

### Fractures

Fractures occurred in 31 CP (18%). The fracture rate was 5.8 fractures per 1000 person-years. Mean age at last follow-up of patients who had fractures was 52.0 ± 14.5 versus 45.5 ± 18.5 years in patients who had no fractures (*P* = .05). Mean follow-up time was 16.8 ± 10.0 years of patients who had fractures versus 18.6 ± 13.7 of patients who had no fractures (*P* = .60, n = 135). Seven of the patients with fractures had osteopenia (70%); 4 had osteoporosis (40%) (at last DXA scan). Eight patients suffered from multiple fractures (26%). The mean number of fractures per patient were 1.5 ± 1.1 (range, 1-6). The fracture types are described in Table 2.

**Table 2.** Frequencies and Site of Fractures

Location	Male (All)	Male (Dutch/Swedish)	Female (All)	Female (Dutch/Swedish)	All Subjects	Dutch/Swedish
Unknown^a^	2 (5%)	1 (5%)/1 (6%)	0 (0%)	0 (0%)/0 (0%)	2 (4%)	1 (4%)/1 (5%)
Femur	2 (5%)	1 (5%)/1 (6%)	0 (0%)	0 (0%)/0 (0%)	2 (4%)	1 (4%)/1 (5%)
Radius	1 (3%)	0 (0%)/1 (6%)	1 (11%)	0 (0%)/1 (33%)	2 (4%)	0 (0%)/2 (10%)
Tibia	2 (5%)	1 (5%)/1 (6%)	1 (11%)	0 (0%)/1 (33%)	3 (7%)	1 (4%)/1 (5%)
Fibula	2 (5%)	0 (0%)/2 (12%)	1 (11%)	1 (17%)/0 (0%)	3 (7%)	1 (%)/2 (10%)
Lower leg (unspecified)	2 (5%)	2 (10%)/0 (0%)	0 (0%)	0 (0%)/0 (0%)	2 (4%)	2 (8%)/0 (0%)
Humerus	1 (3%)	0 (0%)/1 (6%)	2 (22%)	1 (17%)/1 (33%)	3 (7%)	1 (4%)/2 (10%)
Ulna	1 (3%)	1 (5%)/0 (0%)	1 (11%)	1 (17%)/0 (0%)	2 (4%)	2 (8%)/0 (0%)
Clavicle	2 (5%)	1 (5%)/1 (6%)	0 (0%)	0 (0%)/0 (0%)	2 (4%)	1 (4%)/1 (5%)
Cervical vertebrae	3 (8%)	0 (0%)/3 (18%)	0 (0%)	0 (0%)/0 (0%)	3 (7%)	0 (0%)/3 (15%)
Lumbar vertebrae	1 (3%)	1 (5%)/0 (0%)	1 (11%)	1 (17%)/0 (0%)	2 (4%)	2 (8%)/0 (0%)
Long pipebones/vertebrae	17 (46%)	7 (35%)/10 (59%)	7 (78%)	4 (67%)/3 (100%)	24 (52%)	11 (42%)/13 (65%)
Rib	3 (8%)	2 (10%)/1 (6%)	0 (0%)	0 (0%)/0 (0%)	3 (7%)	2 (8%)/1 (5%)
Pelvis	1 (3%)	0 (0%)/1 (6%)	0 (0%)	0 (0%)/0 (0%)	1 (2%)	0 (0%)/1 (5%)
Facial bones/nose	4 (11%)	3 (15%)/1 (6%)	0 (0%)	0 (0%)/0 (0%)	4 (9%)	3 (12%)/1 (5%)
Hand	5 (14%)	4 (20%)/1 (6%)	1 (11%)	1 (17%)/0 (0%)	6 (13%)	5 (19%)/1 (5%)
Foot (calcaneus/toe/unspecified)	5 (14%)	3 (15%)/2 (12%)	1 (11%)	1 (17%)/0 (0%)	6 (13%)	4 (15%)/2 (10%)
Other	18 (49%)	12 (60%)/6 (%)	2 (22%)	2 (33%)/0 (0%)	20 (43%)	14 (52%) / 6 (30%)
Total	37 (100%)	20 (54%)/17 (46%)	9 (100%)	6 (67%)/3 (33%)	46 (100%)	26 (100%)/20 (100%)

Data are presented as n (% of all fractures in this category); 80% of all fractures occurred in males and 57% in the Dutch cohort. Percentages of categories unknown, long pipebones/vertebrae, and other fractures may not add up to 100% because of rounding.

^a^Data were lost during transition of electronic patient files.

Fractures occurred more often in men than women (26% vs. 8%, *P* = .002), and men were less often treated with medication to improve BMD (7% vs. 18%, *P* = .02). A trend was seen toward a higher proportion of fractures in AO CP than in CO CP (23% vs. 12%, *P* = .06). There was no difference in percentage of patients with fractures between Dutch and Swedish patients (18% vs. 17%, *P* = .86).

**Figure 1.**
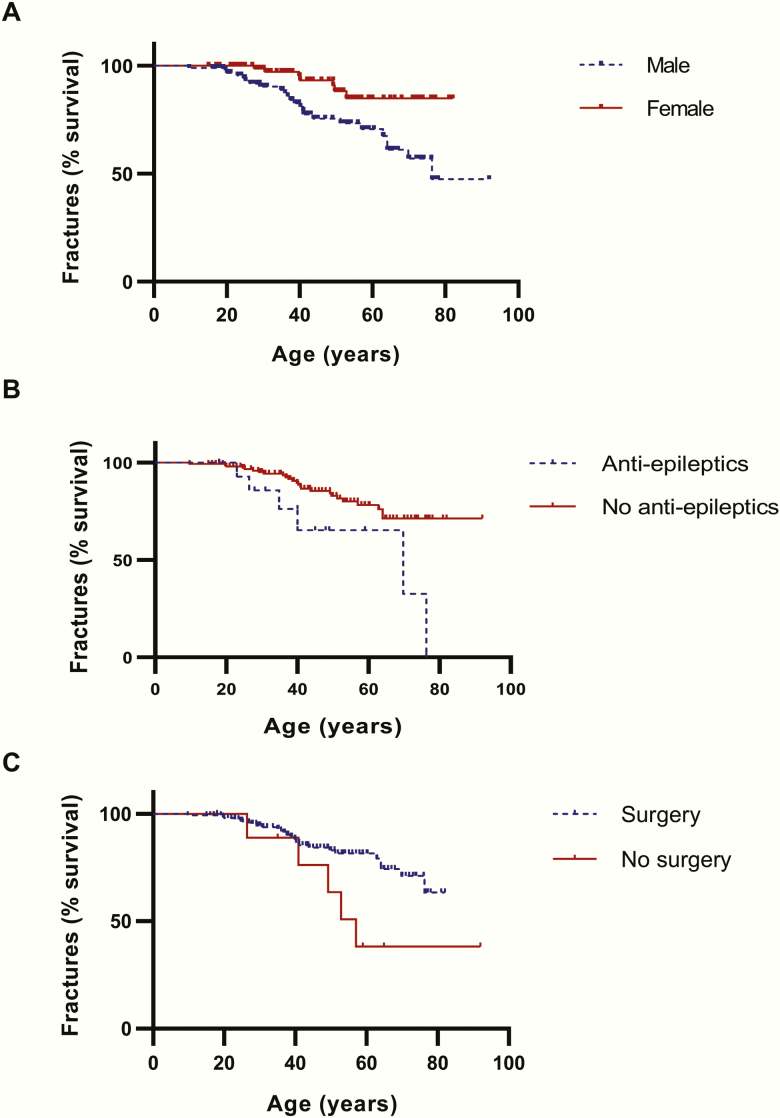
Fracture-free survival (Kaplan-Meier) curves. Multivariable Cox regression model hazard ratios: (A) sex 0.4 (95% CI, 0.2-0.83; *P* = .02), (B) for surgery 0.32 (0.12-0.84; *P* = .02) and (C) for antiepileptics use 2.68 (1.08-6.69, *P* = 0.03) (-2LL χ ^2^ < 0.001, omnibus χ ^2^ 0.001).

Using a univariable logistic regression model investigating fractures, female sex (odds ratio [OR], 0.3; *P* = .004) and previous surgery (OR, 0.2; *P* = .01) were protective factors, whereas medication for epilepsy was identified as a risk factor (OR, 3.6; *P* = .03) (Table 3). Osteoporosis was not a significant independent factor (OR, 2.1; *P* = .21); for osteopenia, a trend was shown (OR, 2.6; *P* = .09). There were 10 patients who never underwent surgery, of which 5 had a fracture; 26 of 165 patients who did have surgery had a fracture. A trend was shown for radiotherapy as well, being protective to fractures (OR, 0.5; *P* = .06). When these independent variables were applied in the multivariable Cox regression model, the following hazard ratios were obtained: for sex 0.4 (95% confidence interval [CI], 0.2-0.8, *P* = .02), for surgery 0.3 (0.1-0.8, *P* = .02) and for antiepileptic medication use 2.7 (1.1-6.7, *P* = .03) (-2LL χ ^2^ < 0.001, omnibus χ ^2^ 0.001). Fracture-free survival curves are depicted in Fig. 1.

**Table 3.** Univariable and Multivariable Logistic Regression Analysis for Determinants of Fractures in CP Survivors

	Univariable Analysis		Multivariable Analysis (Nagelkerke *R*^2^ = 0.18)	
Variables	OR (95% CI)	*P*	OR (95% CI)	*P*
Age at presentation	1.0 (0.99–1.0)	.17		
Female sex	**0.3 (0.1–0.6)**	**.004**	0.3 (0.1–0.7)	**.004**
Adulthood onset disease	0.2 (1.0–4.9)	.07		
Swedish cohort	0.9 (0.4–2.1)	.86		
Surgery	**0.2 (0.1–0.7)**	**.01**	0.1 (0.0–0.6)	**.009**
Radiotherapy	0.5 (0.2–1.0)	.06		
Hypothalamic damage	0.9 (0.4–2.0)	.79		
Visual impairment	1.9 (0.6–5.9)	.26		
Medication for epilepsy	**3.6 (1.2–11.0)**	**.03**	3.0 (0.9–10.0)	.07
Growth hormone deficiency	2.9 (0.6–13.0)	.16		
GHRT	1.7 (0.7–4.3)	.25		
Adrenal axis deficiency	0.9 (0.3–2.3)	.77		
Hydrocortisone dose	1.0 (0.9–1.1)	.95		
Gonadal axis deficiency^a^	2.3 (0.5–10.4)	.28		
TSH deficiency	1.4 (0.3–6.6)	.66		
Diabetes insipidus	1.2 (0.5–2.8)	.62		
BMI	0.9 (0.9–1.0)	.07		
Osteopenia	2.6 (0.9–7.7)	.09		
Osteoporosis	2.1 (0.7–6.4)	.21		
BMD increasing medication	2.1 (0.7–5.8)	.17		

Age at last follow-up not included (violation of assumption of linearity of logit). Bold values represent significant variables (*P* ≤ .05).

^a^Replacement therapy odds ratio, 1.6 (95% confidence interval, 0.7–4.0; *P* = .28)/after exclusion of postmenopausal females odds ratio, 1.3 (95% CI confidence interval, 0.4–4.2, *P* = .62). Hosmer Lemeshow *P* = .81, omnibus χ ^2^ <0.001. No subjects were excluded. Receiver operating characteristic area under the curve, 0.72.

Abbreviations: BMD, bone mineral density; BMI, body mass index; GHRT, GH replacement therapy.

Low BMD values based on either T- or Z-scores was found in 11 patients (65%) with fractures as opposed to 32 patients (42%) without fractures in their history (*P* = .08) (35). Patients who had never been treated with surgery (n = 10) suffered from more fractures than patients who had been operated (50% vs. 16%, *P* = .006). A trend for more fractures was seen in patients who had never received radiotherapy compared with patients treated with radiotherapy (23% vs. 12%, *P* = .06).

### Bone mineral density

**Figure 2.**
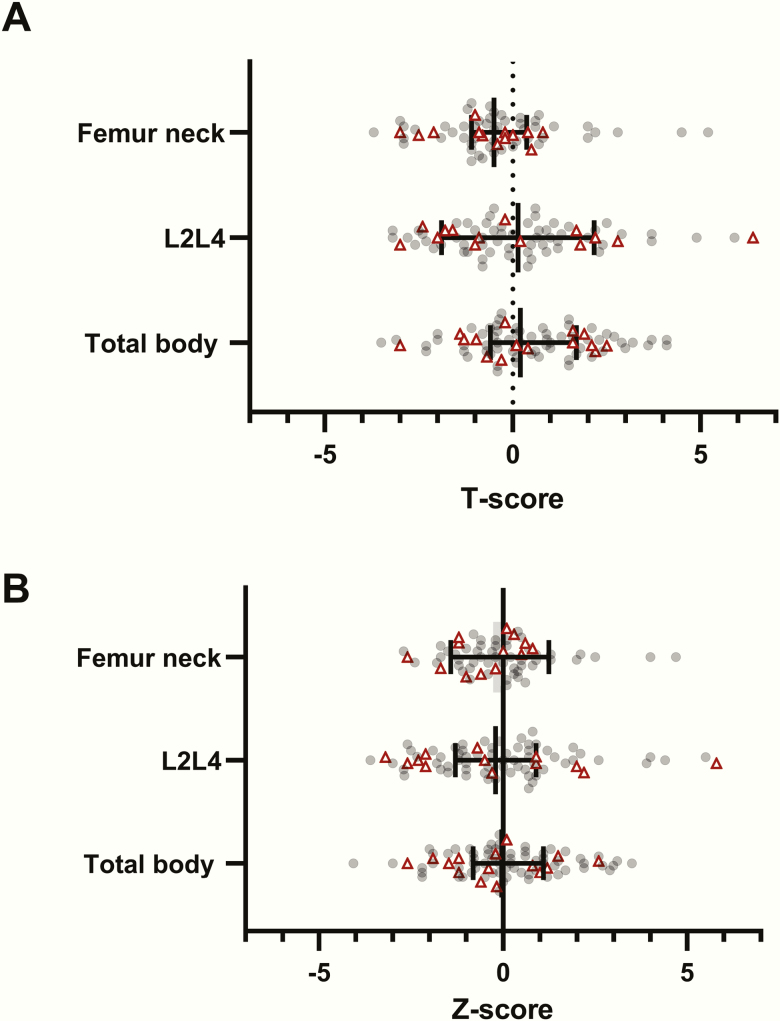
Scatterplot of (A) T- and (B) Z-scores of bone mineral density of the femur neck, L2-L4, and total body in survivors of craniopharyngioma. The error bars express median and quartiles. Every circle or triangle represents a measurement at last dual X-ray absorptiometry scan. The red triangles represent patients who had fractures in the past; the gray circles represent patients who did not have fractures. A wide spread is shown in both patients with and without fractures in the past.

Information on BMD is shown in Table 4. Mean age at last DXA scan is 44.8 ± 18.4 years. Overall mean BMD T- and Z-scores at last DXA scan were in the normal range: for example, for total body, femur neck, and L2-L4, the Z-scores were 0.1 ± 1.5 (range, -4.1 to 3.5), -0.1 ± 1.3 (range, -2.7 to 4.7), and 0.0 ± 2.0 (range, -3.5 to 6.8), respectively (Table 4). However, low BMD in any of these sites was reported in 47 patients (50%) (whereas an SDS of -1 should correspond to a percentage of 16%). Scatter plots of BMD T- and Z-scores are shown in Fig. 2, showing a wide spread of SDS values also in patients with fractures. Osteoporosis occurred in 22 patients (24%) (Table 4). AO CP had a lower BMD of the femur (neck) than CO CP (0.92 ± 0.15 vs. 1.09 ± 0.28, *P* = .01) (Table 4) and a lower mean T-score of the femur (neck) (-0.8 ± 1.2 vs. 0.2 ± 2.1, *P* = .03), but comparable Z-scores. A trend was found for lower Z-score of the femur neck in males than in females (-0.4 ± 1.2 vs. 0.2 ± 1.4, *P* = .08). Of the patients with osteoporosis, 19% has hypothalamic damage, as opposed to 36% of patients without osteoporosis (*P* = .15).

**Table 4.** Bone Mineral Density and Final Height of the Included CP Survivors

		All		Male		Female			CO CP		AO CP		
		Mean (SD)	n	Mean (SD)	n	Mean (SD)	n	*P* Value	Mean (SD)	n	Mean (SD)	n	*P* Value
Age at last DXA^a^		44.8 (18.4)	95	47.1 (19.2)	47	42.6 (17.4)	48	.26	32.9 (14.3)	45	55.6 (14.7)	50	**<.001**
BMI at last DXA		31.3 (5.3)	84	30.9 (4.8)	43	31.8 (5.8)	41	.41	31.1 (5.1)	37	31.6 (5.5)	47	.69
BMD	Total body	1.20 (0.16)	78	1.25 (0.15)	43	1.14 (0.15)	35	**.002**	1.19 (0.17)	37	1.21 (0.15)	41	.72
	Femur	1.04 (0.22)	65	1.00 (0.20)	32	1.07 (0.23)	33	.26	1.11 (0.26)	27	0.98 (0.16)	38	**.03**
	Femur neck	0.99 (0.22)	34	0.95 (0.20)	31	1.03 (0.24)	33	.12	1.09 (0.28)	25	0.92 (0.15)	39	**.01**
	L2-L4	1.24 (0.25)	76	1.32 (0.27)	37	1.17 (0.21)	39	**.01**	1.22 (0.21)	34	1.26 (0.28)	42	.53
T-score	Total body	0.5 (1.7)	79	0.5 (1.8)	44	0.5 (1.6)	35	.85	0.4 (1.7)	37	0.5 (1.7)	42	.87
	Femur	-0.1 (1.5)	63	-0.4 (1.5)	30	0.1 (1.6)	33	.22	0.4 (1.9)	24	-0.4 (1.2)	39	.05
	Femur neck	-0.4 (1.6)	64	-0.8 (1.5)	31	-0.1 (1.6)	33	.09	0.2 (2.1)	24	-0.8 (1.2)	40	**.03**
	L2-L4	0.1 (2.0)	73	0.5 (2.1)	36	-0.2 (1.8)	37	.10	-0.1 (1.6)	31	0.3 (2.3)	42	.46
Z-score	Total body	0.1 (1.5)	84	0.0 (1.6)	45	0.2 (1.3)	39	.57	-0.1 (1.6)	41	0.2 (1.4)	43	.39
	Femur	0.0 (1.4)	65	-0.3 (1.3)	32	0.2 (1.4)	33	.17	0.2 (1.7)	26	-0.2 (1.1)	39	.29
	Femur neck	-0.1 (1.3)	64	-0.4 (1.2)	31	0.2 (1.4)	33	.08	0.2 (1.8)	24	-0.3 (1.0)	40	.20
	L2-L4	0.0 (2.0)	75	0.3 (2.2)	36	-0.3 (1.7)	39	.18	-0.4 (1.6)	33	0.3 (2.2)	42	.10
Final height		172.3 (10.1)	171	178.1 (8.0)	91	165.6 (8.0)	80	**<.001**	172.9 (11.2)	80	171.7 (9.0)	91	.41
Final height SDS		-0.3 (1.2)	171	-0.3 (1.2)	91	-0.3 (1.2)	80	.82	-0.3 (1.4)	80	-0.3 (1.0)	91	.90
Low BMD		47 (50%)	94	25 (53%)	47	22 (47%)	47	.54	21 (49%)	43	26 (51%)	51	.84
Osteoporosis		22 (23%)	94	13 (28%)	47	9 (19%)	47	.33	8 (19%)	43	14 (28%)	51	.31
Osteopenia		43 (46%)	94	21 (45%)	47	22 (47%)	47	.84	18 (42%)	43	25 (49%)	51	.49

Age is given in years. BMD (T- and Z-scores) are given of the first DXA scan. For the raw BMD and BMI values, children were excluded. The minimum age at first DXA scan is 6 years. Data are given as mean (SD); proportions are given as n (%). Low BMD is a T- or Z-score below -1. Osteoporosis is a T-score below -2.5 or a Z-score below -2. Osteopenia is a T-score between -1 and -2.5 or a Z-score between -1 and -2. This is evaluated at 3 locations: L2L4, femur neck, and total body. Patients were also included if data were available at less than 3 locations. Bold values represent significant differences between groups (P ≤ .05).

Abbreviations: AO, adulthood onset; BF%, body fat percentage; BMD, bone mineral density (in g/cm^2^); CO, childhood onset; CP, craniopharyngioma patient; DXA = dual X-ray absorptiometry scan; L2-L4 = located at lumbar vertebral body 2–4; SDS = standardized deviation scores.

^a^n = 95.

First, a univariable analysis for very low BMD/osteoporosis was established: (borderline) significant contributors were age at DXA scan (OR, 1.032 [1.004-1.063], *P* = .03), obstructive sleep apnea syndrome (OR, 2.9; 95% CI, 1.0-8.3; *P* = .05), and hydrocortisone dose (OR, 1.1; 95% CI, 1.0-1.2; *P* = .06). After establishing a multivariable analysis, age (OR, 1.03; 95% CI, 1.00-1.06; *P* = .03) was identified as a contributing independent prognostic factor (35). Hypothalamic damage did not contribute significantly to osteoporosis, nor did it contribute in a multivariable model for osteopenia when adjusted for age, medication to improve BMD and GHRT (OR, 1.6, *P* = .49, model not significant).

### Final height

CP had a final height SDS within the normal range (Table 4). There were no differences in final height SDS between males and females (-0.3 ± 1.2 vs. -0.3 ± 1.2, *P* = .82) or between CO and AO CP (-0.3 ± 1.4 vs. -0.3 ± 1.0, *P* = .90).

## Discussion

Eighteen percent of the CP had suffered from at least 1 fracture during a median follow-up of 16 years. Fractures occurred more often in males than females and males were less often treated with medication intended to improve BMD, which could indicate that males were undertreated. DXA revealed overall mean BMD values in the normal range in our cohort and no differences in low BMD or T- or Z-scores between patients with and without fractures. However, the spread was wide: 46% had osteopenia and 24% had osteoporotic BMD T- or Z-scores.

In our study, osteoporosis was not a predictor of fracture risk. Obviously, there are more factors than only BMD that contribute to fracture risk. In the literature, prior and current exposure to glucocorticoids was associated with an increased fracture risk of substantial importance beyond explanations by BMD (41). Using our logistic regression model for fractures, we did not find any role for pituitary hormonal deficiencies in the increased fracture rates. This is probably because of adequate replacement therapy, as illustrated by replacement rates of 94% for hypogonadotropic hypogonadism. As expected, epilepsy medication accelerated fracture risk, whereas previous treatment with surgery and female sex were protective. Antiepileptic drugs can induce poor balance or clumsiness; certain antiepileptic drugs are associated with an increased rate of bone loss (17), and some may cause hyponatremia, which correlates with fractures and low BMD (42).

The observation that surgery as previous tumor treatment was identified as a “protective” factor was surprising. Only a few patients were not treated with surgery; perhaps these are patients severely affected by comorbidities where surgery could not safely be performed, thereby having an increased risk for fractures as well. Patients with a fracture in their history had a higher mean age, were more often male, and less often had surgery.

Female sex was observed to be a protective factor, which corresponds with the findings of Müller et al. (24). This might be related to the relatively young age of our study population and to a higher BMI in women. In the premenopausal general population, higher frequencies of fractures and lower osteoporosis preventive medication have been described in men, whereas in the postmenopausal general population, female sex is a risk factor for fractures (43–45).

A trend was shown for radiotherapy being protective for fractures. The general aim of the surgeons in treatment of CP have shifted from attempting gross total resection toward aiming at a more limited resection today, which may leave more tumor mass behind and cause the need to additional treatment with radiotherapy. Presumably, the lower risk for fractures observed in our study is therefore explained by a less damaging treatment strategy.

We found a high percentage of fractures in our study because the cumulative fracture incidence in a European population was reported to be 7.8% in men and 9.5% in women (19). In our previous study, no higher risk was found for fractures in our CP cohort (10). This may partly be related to a lack of reliable Dutch reference data, which prompted Wijnen et al. to compare fracture incidence in Dutch patients with Swedish reference data. Fracture rate may differ between populations: it has been reported that Scandinavian countries have higher fracture rates than western Europe (46). Olsson et al. previously showed an increased fracture risk (1).

In a GHD population from the KIMS study (Pfizer International Metabolic Database), fracture rates were reported to be even higher compared with our study (27% in males; 29% in females); no significant differences of fracture prevalence between patients with or without (multiple) other hormonal pituitary deficiencies were found (21). This suggests that GHD itself is an important factor in fracture risk, freestanding from other hormonal pituitary deficiencies. Given the high prevalence of GHD patients in our study, one might expect an even higher a frequency of fractures. Again, a demographic factor could play a role for a higher frequency of fractures, or a registration bias, or compensation of BMD by the protective factor of obesity in CP (47). Obesity occurred in up to 75% of our patients (34) and a trend was shown for BMI as protective factor for fractures in our univariate logistic regression analysis. Gonadal axis hormonal deficiency does not seem to be a major contributor for a higher frequency of fractures, as demonstrated by our results; probably because 94% of premenopausal women and men received hormonal replacement therapy.

The mean BMD at last DXA scan was normal in the total cohort, but low BMD occurred in 50% and osteoporosis in 24% (Table 4). The dispersion was rather large and similar in patients with and without fractures as showed in Fig. 1. BMD T- and Z-scores were similar between genders except for Z-score of the femur neck, which was lower in males (-0.4 ± 1.2 vs. 0.2 ± 1.4, *P* = .08). Sex or GHD were not a contributing factor in our logistic regression model for osteoporosis.

Final height in our patients was not impaired: the mean SDS was -0.3 ± 1.2. It was also similar between AO CP and CO CP, and between genders. Apparently, GHRT had been sufficiently administered to these children. Indeed, 87% of patients with CO CP and GHD received GHRT. This is different from the findings of Kendall-Taylor et al., who did find for AO CP a significant difference in height (although it was on average only 3 cm or 0.6 SDS) (19). Perhaps the large size of their European cohort made it possible to find smaller differences, or there may be regional differences in treatment of CP, particularly the age at which GHRT is started.

Because of the retrospective cross-sectional design, there are some limitations of our study. Factors that may have contributed to a possible selection bias are the size and weight limitations of DXA scanners (48), DXA scans were performed based on individual care plan decided by the insight of the clinicians. All types of DXA scanners were included, whereas measurements by different DXA scanners have shown intra-individual variability (49). However, agreement between all DXA devices is high (50). By using age- and sex-matched SDS and applying generally used cutoffs to identify osteopenic or osteoporotic BMD values, we tried to limited the impact on our data. In our logistic regression model for osteoporosis, scanner type was not a contributing factor. Because of the retrospective design, relevant data on duration of GHRT, smoking, alcohol use, dietary intake, fractures of parents, physical activity, and level of 25-hydroxyvitamin D were lacking. The percentage of fractures could be underestimated because of a reporting bias. Still, since studies on bone health in CP are so limitedly available, our study provides more insight and should create awareness for the importance of attention to bone health in CP.

This is the first study to date that investigated contributory factors for fractures in CP and that describes bone health in a cohort existing of both CO and AO long-term survivors, with a large sample size. With this study, we hope to raise more awareness for the evaluation of bone health. The decision to either or not perform a DXA scan is important in this respect, although DXA scans alone do not evaluate the quality of the bone or fracture risk sufficiently. Fracture rate could also be compromised by, for example, neurological defects. Nevertheless, clinicians should consider treatments that increase bone health, especially in males.

In conclusion, our cohort of CP showed a high frequency of fractures. Although men had a higher frequency of fractures, they received less frequent BMD-increasing treatments. Pituitary hormone replacement therapy in our cohort overall seemed adequately managed because pituitary hormonal deficiencies did not contribute significantly to fracture risk in our logistic regression model. Also of interest was our finding that osteoporotic BMD values did not predict the fracture risk. Our study suggests the importance of regularly evaluating fracture risk in every CP. We advise to take other risk factors than BMD into account, and suggest earlier evaluation of bone health increasing medication, especially in men.

## Abbreviations

AO: adulthood onset
BMD: bone mineral density
BMI: body mass index
CO: childhood onset
CP: craniopharyngioma patient
DXA: dual X-ray absorptiometry
GHD: growth hormone deficiency
GHRT: GH replacement therapy
OR: odds ratio
SDS: standardized deviation score

## Additional Information

***Disclosure Summary:*** The authors have nothing to disclose.

***Data availability:*** Restrictions apply to the availability of data generated or analyzed during this study to preserve patient confidentiality or because they were used under license. The corresponding author will on request detail the restrictions and any conditions under which access to some data may be provided.
